# Does the implementation of an incentive scheme increase adherence to diabetes guidelines? A retrospective cohort study of managed care enrollees

**DOI:** 10.1186/s12913-023-09694-z

**Published:** 2023-06-29

**Authors:** Renato Farcher, Sereina M. Graber, Nicole Thüring, Eva Blozik, Carola A. Huber

**Affiliations:** 1 Department of Health Sciences, Helsana Group, P.O. Box, Zürich, Switzerland; 2 Institute of Primary Care, University of Zürich, University Hospital Zürich, Zürich, Switzerland; 3 Department of Managed Care, Helsana Group, P.O. Box, Zürich, Switzerland

**Keywords:** Pay-for-performance, Primary care, Quality measurement, Claims data, Guideline adherence, Diabetes mellitus

## Abstract

**Background:**

A novel incentive scheme based on a joint agreement of a large Swiss health insurance with 56 physician networks was implemented in 2018. This study evaluated the effect of its implementation on adherence to evidence-based guidelines among patients with diabetes in managed care models.

**Methods:**

We performed a retrospective cohort study, using health care claims data from patients with diabetes enrolled in a managed care plan (2016–2019). Guideline adherence was assessed by four evidence-based performance measures and four hierarchically constructed adherence levels. Generalized multilevel models were used to examine the effect of the incentive scheme on guideline adherence.

**Results:**

A total of 6′273 patients with diabetes were included in this study. The raw descriptive statistics showed minor improvements in guideline adherence after the implementation. After adjusting for underlying patient characteristics and potential differences between physician networks, the likelihood of receiving a test was moderately but consistently higher after the implementation of the incentive scheme for most performance measures, ranging from 18% (albuminuria: OR, 1.18; 95%-CI, 1.05–1.33) to 58% (HDL cholesterol: OR, 1.58; 95%-CI, 1.40–1.78). Full adherence was more likely after implementation of the incentive scheme (OR, 1.37; 95%-CI, 1.20–1.55), whereas level 1 significantly decreased (OR, 0.74; 95%-CI, 0.65 – 0.85). The proportions of the other adherence levels were stable.

**Conclusion:**

Incentive schemes including transparency of the achieved performance may be able to improve guideline adherence in patients with diabetes and are promising to increase quality of care in this patient population.

**Supplementary Information:**

The online version contains supplementary material available at 10.1186/s12913-023-09694-z.

## Background

Pay-for-performance (P4P) is a financial incentive to improve the quality of care, where health care providers were remunerated for achieving selected quality measures [1, 2]. Given the increasing prevalence of diabetes, several countries implemented P4P schemes for incentivizing provision of quality of diabetes management in health care [3–5, 1, 6]. The positive effect of P4P scheme on the quality of diabetes care was demonstrated in few studies [7–10]. For example, several UK studies reported improvements in diabetes care in general practices after the implementation of the Quality and Outcomes Framework (QOF), the national contractual P4P scheme [9–11]. Kontopantelis et al. [11] showed an overall increase in the quality of diabetes care after the first year of the implementation by using a quality of care score. Also, Vaghela et al. [10] evaluated the effect of QOF on clinical quality measures and revealed improvement in blood pressure, HbA1c and in total cholesterol level. A recent Canadian study investigating the effect of an incentivized diabetes management program targeting diabetes process measures demonstrated that patients enrolled in a P4P program had a significant higher probability of receiving diabetes related services [7]. However, despite the existing studies indicating positive effects of incentive schemes, the empirical evidence on the quality of diabetes care remains inconclusive [12–18]. A recent published study for Switzerland which examined the effects of financial incentives on clinical and process quality measures showed no effect on the assessment of HbA1c tests and on the achievement of targeted blood pressure level [18]. Insignificant results on clinical quality measures were also reported in Chien et al. [16] which evaluated the effect of a P4P program in patients who were enrolled in a managed care plan in the state of New York. A Canadian study also revealed no significant differences in primary care visits, continuity of care, hospitalization, emergency visits nor in health care spending before vs. after the introduction of an P4P scheme for primary care physicians [12]. The inconclusive effect of the P4P schemes is not only limited to diabetes care. Several studies examined the effect of P4P scheme on outcomes of other chronic conditions and also found heterogenous effects in various health care settings such as inpatient [19, 20] and primary care [21, 20].

In Switzerland, a novel incentive scheme implemented in established contracts between one of the largest basic mandatory health insurances and 56 physician networks was introduced in 2018. Physician networks consist of independent physicians and physicians of health maintenance organizations (HMO). The physicians in the network commit to a network specific quality standard (e.g. quality certification; quality circles) and to an incentive system that includes economic and qualitative elements. The Swiss Federal Law on Health Insurance (KVG/ LAMal) allows physician networks and health insurances to negotiate cooperation agreements for patients enrolled in managed care models. The novel type of agreement evaluated in the present paper included incentives in two ways: benchmarking of the performance level of each physician network related to the proportion of their patients with full adherence to the combination of all four performance measures in the previous year, and financial incentives in form of additional payments for the best performing physician networks. Best performing was defined as 40% of the physician networks on the benchmark, who demonstrated the highest proportion of patients with full adherence. Information about the cooperation agreements including the incentive scheme between the physician networks and the health insurer is not publicly available. However, the incentives are structured the same for all physician networks.

The aim of this study was to evaluate the effect of a novel and broadly implemented incentive scheme in Switzerland on the guideline adherence of patients with diabetes enrolled in the underlying managed care models.

## Methods

### Study design and study population

We performed a retrospective cohort study using anonymized claims data of patients with diabetes who had mandatory health insurance and were enrolled in a managed care plan with Helsana, a leading health insurance company in Switzerland, from 2016 to 2019. In contrast to standard care plans, patients with managed care plans chose a gatekeeper who coordinates the treatment process. This study included enrollees in contracted family doctor based managed care models, in which insured persons have a defined primary care physician as first provider of care who also coordinates further care if necessary. In these managed care models, a network of physicians (individual physicians or health maintenance organizations) concludes contracts with the health insurer, which regulate, among other things, the requirements for quality measures and compensation.

Patients with diabetes were identified by the prescribed medication, which included information about active ingredients defined by the Anatomical Therapeutic Chemical (ATC) code published by the World Health organization (WHO) [25]. Patients with diabetes were identified by at least one oral blood glucose-lowering drug without insulin (ATC code A10B) or insulin (ATC code A10A) prescriptions for two consecutive years. The claims data contained information about gender, age, chosen insurance plan (standard or managed care model), cost of medication and cost of in- and outpatient care of the enrollees. Furthermore, it contained diabetes specific information such as frequency of received laboratory tests or specialist visits.

To evaluate the effect of the incentive scheme on guideline adherence, we included individuals who were continuously insured in a family doctor based managed care model and did not change the physician network from 2016 to 2019. Patients who were younger than 18 or older than 85 years old, pregnant, living in a nursing home or who died during the observation period were excluded.

### Diabetes performance indicator

Diabetes guideline adherence reflecting the quality of diabetes care was assessed by two diabetes performance indicators, which were developed and presented in a previous study [26]. The indicators assess not only the adherence to single recommended diabetes tests, but also the adherence to the overall diabetes guidelines and are described as follows:

1. Evidence-based single performance measures, extracted from international medical recommendations for diabetes care [27], included the frequency of HbA1c test, lipid profile test, nephropathy status test and the frequency of ophthalmologist visits. The assessment of the lipid profile test contained the annual frequency of the total cholesterol test, HDL cholesterol test, LDL cholesterol test, and triglycerides test. The assessment of the nephropathy status test comprised the annual frequency of the serum creatinine test, and the albuminuria test. The performance measure for HbA1c test was considered as achieved if at least two tests were performed in one year. The performance measure for the ophthalmologist visit was considered as achieved if a consultation was carried out once a year.
2. Adherence levels, which were based on international medical guidelines and introduced by Huber and colleagues [26]. In total, four levels of adherence were constructed to analyze the distribution of the degree of guideline adherence in patients with diabetes. The adherence levels were built up hierarchically and cumulatively. A higher level of adherence was associated with better adherence. Patients were exposed to adherence level 1 if they received at least two HbA1c test in one year. Patient were exposed to adherence level 2 if they fulfilled the criteria of adherence level 1 (definition see above) and received in addition at least one lipid profile test in one year. To be exposed to adherence level 3 the patient fulfilled the criteria of adherence level 2 and additionally received a nephropathy status test in one year. Patients were exposed to the full guideline adherence (level 4) if they fulfilled the criteria of adherence level 3 and had an ophthalmologist visit in one year. Patients with diabetes who received only 1 or no HbA1c test in one year were exposed to adherence level 0.

### Statistical analysis and outcome

We used descriptive statistics to analyze the frequency and the proportion of patients with diabetes who had fulfilled the performance measures and the guideline adherence levels in each year (2016–2019). To evaluate the effect of the incentive scheme on guideline adherence, we estimated generalized multilevel models to predict the probability of receiving a test of the performance measure as well as the probability of the exposure to the adherence level before versus after the implementation period and between the sample years. The period before the implementation comprised the years 2016 and 2017 and the period after the implementation the years 2018 and 2019.

For each performance indicator and adherence level we calculated a separate model, extracting the adjusted odds ratios (OR) and the corresponding 95% confidence intervals (CI). The models used age, sex, medication and in- and outpatient costs as fixed effects. To account for variation within the physician networks as well as within insured individuals, the models included a corresponding nested effect as random intercepts. To estimate the differences between specific years within the described multilevel models, we applied planned non-orthogonal contrasts (post-hoc adjusted for multiple testing). Planned contrasts refer to when testing specific predetermined comparisons, in our case the specific comparisons between the years. Non-orthogonal refers to non-independent comparisons (i.e. same years are used in multiple comparisons), and thus multiple testing based on the same sample, leading to an inflated familywise error rate, which eventually requires adjusted test-statistics. We assigned the contrasts for the different time groups as follows: Contrast 1: 2016/2017 vs. 2018/2019, Contrast 2: 2016 vs. 2017, Contrast 3: 2017 vs. 2018, Contrast 4: 2018 vs. 2019. Data were analyzed using R, version 3.5.0 (R Foundation for Statistical Computing, Vienna, Austria [28]). Multilevel models were ran using the lme4 package [29] and adjustments for multiple testing were done using the multcomp package [30].

### Ethics approval and informed consent

The study complied with the national ethical and legal regulations. The study used retrospective, pre-existing, anonymized and de-identified routine administrative health care claims data. The data was anonymized and de-identified before the analysis. Thus, according to the Swiss Federal Law on human research [31] and the local ethics committee (Kantonale Ethikkommission Zürich) an ethical approval and seeking informed consent of patient was not required.

## Results

After applying the inclusion and the exclusion criteria, the final study population comprised 6′273 patients with diabetes who were enrolled in a managed care model and did not change the physician network from 2016 to 2019 for our analysis. Patient characteristics are shown in Table 1. About 40% of the patients were women and over 60% were between 60 to 79 years old. The highest health care cost was reported for the outpatient care followed by the cost for medication and inpatient care. The health care cost increased from 2016 to 2019 for all cost groups.

**Table 1:** Patient characteristics of the diabetes cohort before and after implementation of the incentive scheme

	**Before implementation of incentive scheme**				**After implementation of incentive scheme**			
	**2016**		**2017**		**2018**		**2019**	
**Total**	6′273	25%	6′273	25%	6′273	25%	6′273	25%
**Sociodemographics**								
Female	2′531	40.3%	2′531	40.3%	2′531	40.3%	2′531	40.3%
Age group (years)								
18–39	143	2.3%	126	2.0%	110	1.8%	103	1.6%
40–59	1′527	24.3%	1′389	22.1%	1′275	20.3%	1′132	18.0%
60–79	4′157	66.3%	4′134	65.9%	4′084	65.1%	4′054	64.6%
> 79	446	7.1%	624	9.9%	804	12.8%	984	15.7%
**Total health care cost (CHF)**								
Outpatient cost (SE)	4′250.3	(5′275.5)	4′527.1	(6′040.93)	4′605.9	(6′361.7)	5′204.5	(7′888.8)
Medication cost (SE)	2′724.4	(3′922.0)	2′900.2	(4′114.18)	3′083.4	(5′559.4)	3′221.9	(5′387.0)
Inpatient cost (SE)	1′639.5	(5′5598.9)	1′690.8	(6′170.6)	1′787.8	(5′663.7)	1′986.5	(6′410.0)

*Abbreviations*: *CHF* Swiss Francs, *SE* Standard Error

Table 2 presents the number and proportion of diabetes patients who fulfilled performance measures and exposed adherence levels from 2016 to 2019. The presented descriptive statistics are based on raw data and thus, are not adjusted for potential confounders.

**Table 2:** Performance measures and adherence levels before and after implementation of the incentive scheme

	**Before implementation of incentive scheme**				**After implementation of incentive scheme**			
**Performance indicators**	**2016**		**2017**		**2018**		**2019**	
**Performance measures**								
Biannual HbA1c test	5′061	80.7%	5′027	80.1%	5′036	80.3%	5′086	81.1%
Annual total cholesterol test	4′423	70.5%	4′374	69.7%	4′514	72.0%	4′484	71.5%
Annual HDL cholesterol test	4′043	64.5%	4′061	64.7%	4′288	68.4%	4′305	68.6%
Annual LDL cholesterol test	1′543	24.6%	1′526	24.3%	1′580	25.2%	1′559	24.9%
Annual triglycerides test	4′235	67.5%	4′221	67.3%	4′363	69.6%	4′352	69.4%
Annual lipid profile test (total cholesterol/HDL/LDL and triglycerides)	4′234	67.5%	4′218	67.2%	4′358	69.5%	4′350	69.3%
Annual serum creatinine test	5′173	82.5%	5′211	83.1%	5′356	85.4%	5′363	85.5%
Annual albuminuria test	3′235	51.6%	3′335	53.2%	3′339	53.2%	3′446	54.9%
Annual nephropathy status test (serum creatinine and albuminuria)	3′008	48.0%	3′116	49.7%	3′155	50.3%	3′239	51.6%
Annual visit to an ophthalmologist	4′183	66.7%	4′295	68.5%	4′436	70.7%	4′464	71.2%
**Adherence level**								
Level 4 (full adherent)	1′648	26.3%	1′699	27.1%	1′837	29.3%	1′879	30.0%
Level 3	583	9.3%	543	8.7%	501	8.0%	504	8.0%
Level 2	1′432	22.8%	1′378	22.0%	1′412	22.5%	1′410	22.5%
Level 1	1′398	22.3%	1′407	22.4%	1′286	20.5%	1′293	20.6%
Level 0 (non-adherent)	1′212	19.3%	1′246	19.9%	1′237	19.7%	1′187	18.9%

*Abbreviations*: *HbA1c* Hemoglobin A1c, *HDL* High-density lipoprotein, *LDL* Low-density lipoprotein

Level 0: < 2 HbA1c tests within one year; Level1: ≥ 2 HbA1c tests within one year; Level 2: Level 1 and annual lipid profile; Level 3: Level 2 and annual nephropathy status; Level 4: Level 3 and visit to an ophthalmologist within one year

For most single performance measures, the proportion of patients with diabetes who received at least one test was slightly higher after the implementation of the incentive scheme. For example, in 2016 and 2017, the annual HDL cholesterol test was performed in around 65% of the patients, whereas in 2018 and 2019, almost 70% received a test. For the performance measure of nephropathy status, in 2016 and 2017, 82.5% respectively 83.1% of the patient received an annual serum creatinine test, compared to 85.4% in 2018 and 84.5% in 2019. Moreover, in 2016 and 2017 66.7% respectively 68.5% of the patients visited an ophthalmologist, compared to 70.7% in 2018 and 71.2% in 2019.

The proportion of patients with diabetes who were fully adherent (level 4) increased in the years after implementation. While the proportion of patients who were exposed to adherence level 3 and 1 decreased in the years after the implementation, there was no notable difference in the exposure to adherence level 2 and 0 over the four years.

**Fig. 1:**
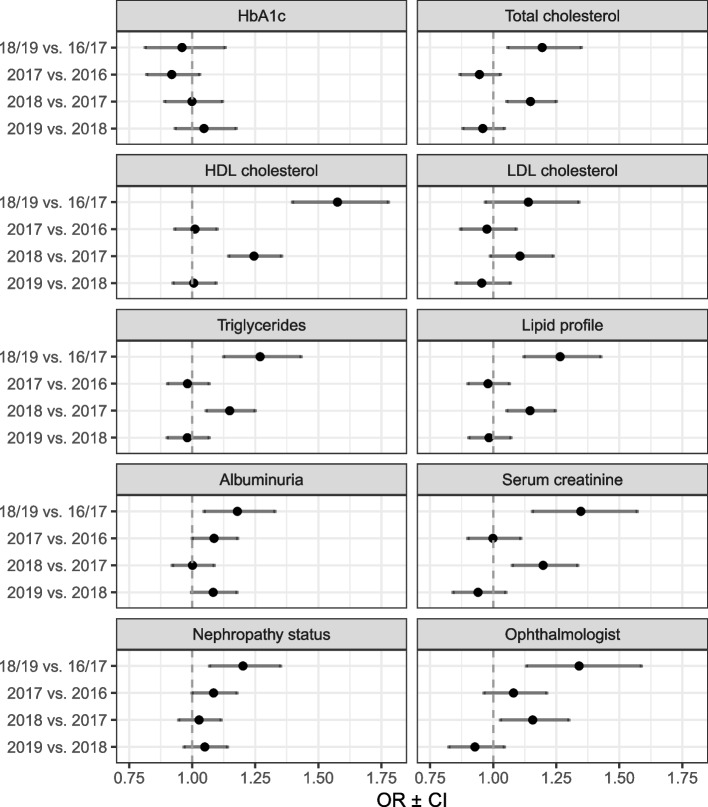
Predicted probability of receiving diabetes performance measures after implementation of incentive scheme. Abbreviation: OR, odds ratio; CI, confidence interval; HbA1c hemoglobin A1c; HDL, high-density lipoprotein; LDL, low-density lipoprotein

The likelihood of receiving a test of the single performance measures, as a function of before versus after the implementation period and between the single sample years, is shown in Fig. 1.

For 8 out of 10 single performance measures, the likelihood to receive a test of the performance measures was significantly higher after the implementation period as compared to before. Regarding lipid measures, the annual HDL test showed with 58% (OR, 1.58; 95% CI, 1.40–1.78) the highest increase after the implementation followed by the annual triglyceride test (OR, 1.27; 95% CI, 1.12–1.43), the annual total lipid profile test (OR, 1.26; 95% CI, 1.13–1.43) and the annual total cholesterol test (OR, 1.19; 95% CI, 1.06–1.35). For the performance measure of the nephropathy status, patients were 35% (OR, 1.35; 95% CI, 1.16–1.57) and 20% (OR, 1.20; 95% CI, 1.07–1.35) more likely to receive an annual creatinine test respectively an annual total nephropathy status test after the implementation. For the annual albuminuria test, the likelihood was 18% (OR, 1.18; 95% CI, 1.05–1.33) higher after the implementation period. For the ophthalmologist visit, patients were 34% (OR, 1.34; 95% CI, 1.13–1.59) more likely to have an ophthalmologist visit within one year after the introduction of the incentive scheme.

Moreover, the likelihood to receive a test was significantly higher in the year 2018 than 2017 for 6 out of the 10 performance measures: the annual total lipid profile test, annual HDL cholesterol test, annual triglyceride test, annual total cholesterol test, annual creatinine test and for the annual ophthalmologist visit. With exception of the total nephropathy status test and the albuminuria test, no significant differences were found in the likelihood to receive a test of the other performance measures between the years 2016 vs. 2017 and 2018 vs. 2019. The detailed estimations are presented in Additional file 1.

**Fig. 2:**
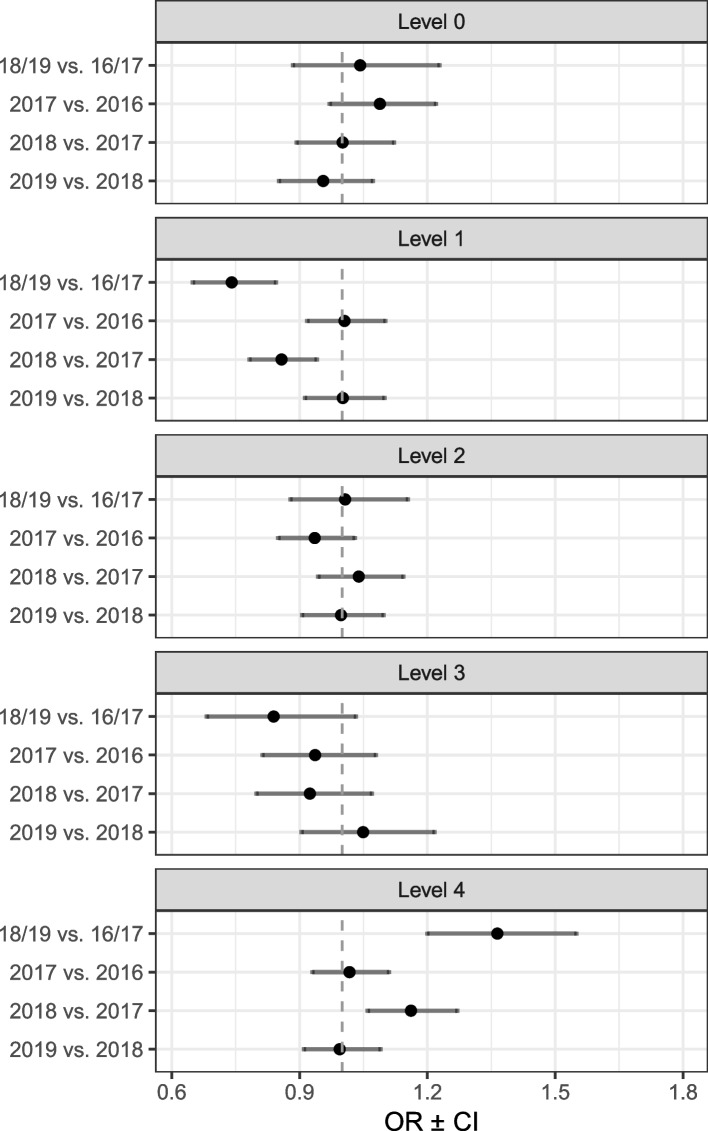
Predicted probability of achieving diabetes adherence levels after implementation of incentive scheme. Abbreviation: OR, odds ratio; CI, confidence interval; HbA1c hemoglobin A1c; HDL, high-density lipoprotein; LDL, low-density lipoprotein. Level 0: < 2 HbA1c tests within one year; Level1: ≥ 2 HbA1c tests within one year; Level 2: Level 1 and annual lipid profile; Level 3: Level 2 and annual nephropathy status; Level 4: Level 3 and visit to an ophthalmologist within one year

The likelihood of achieving diabetes guideline adherence in terms of performance levels, as a function of the before and after the implementation period and between the single sample years, is shown in Fig. 2.

Patients with diabetes were 37% (OR, 1.37; 95% CI, 1.20–1.55) more likely to be exposed to full guideline adherence (level 4) after the implementation period. No significant differences were found in the likelihood to be exposed to adherence level 3, 2 and 0. For adherence level 1, the likelihood of exposure was 26% (OR, 0.74; 95% CI, 0.65–0.84) lower after the implementation period.

Moreover, the likelihood to be exposed to full guideline adherence (level 4) was significantly higher in 2018 compared to 2017, whereas again for adherence level 1 the likelihood of exposure decreased at the same time. The remaining individual year comparisons showed no significant differences for the performance levels. The detailed estimations are presented in Additional file 2.

## Discussion

This study evaluated a large-scale implementation of incentive schemes based on diabetes guideline adherence in the mandatory health insurance system in Switzerland. The implementation of the incentive scheme illustrates a collaborative approach between the health insurance and health care providers to improve the quality of diabetes care and contributes to the ongoing discussion about quality of care in patients with chronic diseases. After implementation of the incentive scheme, patients with diabetes were significantly more likely to receive recommended tests related to prevention of long-term complications as compared to the period before. Moreover, patients with diabetes were 37% more likely to benefit from full guideline adherence i.e., more likely to receive all tests of the single performance measures. At the same time, patients were significantly less likely to be exposed to level 1 and therefore less likely to receive a minimum of 2 HbA1c test per year after the implementation. There were no significant changes in other levels. These results can be explained by the patient flow from level 1 towards level 4, which was the most frequent observed patient flow (results not shown). The raw descriptive statistics (Table 2) show only minor improvements in the distribution of patients who received guideline-based performance measures after versus before the implementation, however, those results are not adjusted for underlying patient characteristics such as age, sex, health status and potential differences between physician networks, which all impact the testing distributions.

The moderate but consistent positive effect of the present incentive scheme on guideline adherence is in line with prior evaluation studies of diabetes process measures [7, 8]. For example, Thavam and colleagues evaluated the effect of incentivized diabetes management on process measures in Ontario, Canada, and demonstrated that patients with diabetes had a higher probability to receive diabetes related services (e.g., lipid test) when patients were treated in an incentive-based diabetes management [7]. A further study examined the effect of the P4P scheme on diabetes guideline adherence in Taiwan. They also used performance measures as proxies for guideline adherence and found a significant improvement in recommended tests among patients enrolled in a P4P program [8].

There are several explanations which can be attributed to the positive effect of the incentive scheme on guideline adherence in the evaluated physician networks. First, the transparency and comparability of the achieved performance levels on the benchmark might have increased the competitiveness between the physician networks to perform better than their colleagues. Second, quality in diabetes care is a continuously recurring topic in health care. By setting the focus of the performance indicators on diabetes, the physician networks might have taken the opportunity to foster quality efforts on diabetes care. Third, high-performing physician networks receive a small amount of an additional payments. Despite the small amount, the financial incentive might have provided additional motivation for better performance.

Some P4P-studies revealed a stronger effect size compared with our effect sizes which might be explained by structural differences in the health care system, coverage and access of care, role, attitude and working method of health service providers [8]. Interestingly, our study results identified two tests of the single performance measures (HbA1c, LDL), which did not significantly increase after the implementation period. For the HbA1c test, the finding is in line with studies which showed low adherence [32, 33, 17] or no effects [17] on the test frequency of HbA1c. This finding is astonishing and emphasizes the need for qualitative research to better understand why primary care physician do not perform this highly recommended test. The comparison to the annual HDL cholesterol tests suggests that physicians in Switzerland might prefer the calculation of the ratio of total and HDL cholesterol over the single testing of LDL cholesterol. Another explanation might be the underlying reimbursement system for laboratory testing, where the LDL test is not remunerated as a single service.

Additionally, our findings showed the strongest effect of the incentive scheme in most process measures in the first year of the implementation compared to the following year. This is also in line with the observation of other studies investigating the effect of incentive-based diabetes programs on recommended performance measures [5, 1, 11]. The increase in guideline-based performance measures at the beginning of the implementation might reflect an initial extra effort of the physician.

Another important discussion point is the impact of these performance measures on the improvement and strengthening of integrated care. Since the Swiss health care system (according to the KVG) does not provide reimbursement for coordination services, the budget agreed between Helsana and the physician networks in the managed care contracts is an essential basis for the physician networks to finance, implement and reimburse integrated chronic care programs for diabetics [34]. Especially through the introduction of the "diabetes performance indicators" by Helsana, the networks were confronted with the need to deal with integrated care and diabetes management [35].

The study has some notable strengths and limitations. The main strength of the study is the use of health care claims data covering comprehensive information on a large cohort of patients with diabetes. Health care claims data is practice-based and a reliable source of information. Furthermore, the country specific health care setting including explicit contracts between health insurance and physician networks allowed us to clearly identify the implementation time and to examine the effect for a homogenous group of patients enrolled in a specific managed care health plan over time. With 10 performance measures we used a more comprehensive set to examine the effect of the incentive scheme than other studies. In addition, we investigated the effect of the implementation on four constructed adherence levels to gain more insight on the distribution of guideline adherence.

The study has also some limitations. Over the years, the progression of diabetes and other unmeasured comorbidities increased and thus the medical need of the patient. Therefore, the higher likelihood of receiving a laboratory test or being exposed to full guideline adherence after the implementation might be attributed to the natural course of the disease. In order to control for a potential progression effect, we additionally included general health status in our models by using health care costs as a proxy.

Further, it is important to state that the applied study design does not allow any causal interpretation of the effects. However, the models included several potential confounders and despite increasing political discussion on quality of care, to our knowledge, there was no other large-scale diabetes intervention implemented during the analyzed period, which might have influenced the presented outcomes. Therefore, we strongly assume that the examined effect was associated with the implemented incentive scheme and results from a more sensitized behavior of primary care physicians. Furthermore, the Swiss health care claims data do not include information on diagnosis or clinical parameters. Thus, we used previously prescribed medication to identify patients with diabetes. The approach, however, is an established method for the identification of chronic conditions when using claims data [36–38]. In our study we did not distinguish type 1 and type 2 diabetes. However, by excluding patients under the age of 18, we assume that we excluded the majority of patient with type 1 diabetes. Due to lack of information in the data, our estimation did not control for risk factors of diabetes, the duration of diabetes, health behavior and other confounders, which might influence the outcome. In addition, even though the population to generalize for already comprises a rather specific group of chronically ill patients (diabetic patients), where we would not expect a strong selection bias due to continuous insurance coverage, it cannot be ruled out with certainty. Another limitation is that our evaluation was based on the years 2016 to 2019, which reflects only short time effects of the implemented incentive scheme. A Swiss study on long-term effects of financial incentives for general practitioners on two diabetes quality indicators suggests that an observational period of 1 year might be too short to capture the full effect of such an intervention [17].

The present study has some implications for practice and future research. Given the short time-period of the evaluation, future research needs to focus on the evaluation of the long-term effect of the incentive scheme. Research should also address the potential negative effect of the monitoring of the performance indicators. For example, physicians might focus only on the achievements of the performance level instead of focusing on the comprehensive diabetes care including comorbidity care. Further research is also needed to understand the mechanisms between performance measures and health outcomes in patient with diabetes [39–41] and how the effects can be positively enhanced. Additionally, other performance measures reflecting the guideline adherence in patients with further chronic diseases should be implemented and evaluated when incentive-based schemes were introduced.

In practice, it is likely that the positive effect on guideline adherence increases the acceptance of the performance indicators in the general practice and makes the treating physician more sensitive for irregular and insufficient assessment of diabetes quality measures. Furthermore, we assume a positive spillover effect on diabetes care of patients who are not enrolled in a contracted family doctor based managed care model but are treated by an incentivized physician. Spillover effect on non-exposed enrollees were also observed in other studies [8, 18]. Despite the promising effects on guideline adherence, there are potential undesirable effects in practice which have to be addressed. Physicians who are exposed to the incentive scheme might crowd out intrinsic motivation to provide comprehensive care and adapt a behavior which focuses primarily on the incentivized and maybe not on relevant performance measures. Moreover, incentivized physicians tend to attract patients who are more likely to achieve the performance indicators (e.g. compliant patients) and raise the risk of a selection bias. However, we strongly assume that these undesirable effects are widely negligible for the presented incentive scheme. The agreements between health insurance and service providers have been shown to be constructive and expedient with respect to quality effort in health care. The financial incentive is small and is in addition to the standard rates that apply to all providers. Furthermore, the membership of the physician network implicates that physicians have to fulfill network specific quality standard (e.g. quality certification; quality circles) and commit to an incentive system that includes economic and qualitative elements. Apart from the presented performance indicator for diabetes, it is important to continuously implement and develop further performance indicators in order to comprehensively assess and improve the quality of health care in Switzerland.

## Conclusion

This evaluation study shows that the implementation of an incentive scheme including transparency of the achieved performance in the context of agreements between health insurance and physician networks can significantly improve guideline adherence reflecting the quality of care among patients with diabetes.

## Supplementary Information

**Additional file 1.** Predicted probability of achieving diabetes adherencelevels after implementation of incentive scheme. *N*=6273.

**Additional file 2.** Predicted probability of receiving diabetesperformance measure after implementation of incentive scheme. *N*=6273.

## Data Availability

There is restriction to the public availability of the analyzed dataset. They are part of the confidential Helsana health insurance claims database and were used under license of the current study. Additional information is available from the corresponding author on request.

## Abbreviations

ATC: Anatomical Therapeutic Chemical
CI: Confidence intervals
QOF: Quality and Outcomes Framework
HbA1c: Hemoglobin A1c
HDL: High- density lipoprotein
HMO: Health maintenance organizations
KVG/ LAMal: The Swiss Federal Law on Health Insurance
LDL: Low-density lipoprotein
MPA: Medical practice assistant
OR: Odds ratio
P4P: Pay-for-performance
UK: United Kingdom
WHO: World Health Organization
