# Supplementary material for: Does the implementation of an incentive scheme increase adherence to diabetes guidelines? A retrospective cohort study of managed care enrollees
Source: BMC Health Serv Res. 2023 Jun 29;23:707. doi: 10.1186/s12913-023-09694-z (PMC10308744; doi:10.1186/s12913-023-09694-z)
Supplement: Supplementary file 1 — Additional file 1. Predicted probability of achieving diabetes adherencelevels after implementation of incentive scheme. N=6273. [file 12913_2023_9694_MOESM1_ESM.docx]

**Additional File 1**

Additional file 1: Predicted probability of achieving diabetes adherence levels after implementation of incentive scheme

| **Adherence Levels** |  |  |  |
| --- | --- | --- | --- |
| **Level 4** | **OR** | **95% CI** | |
| 18/19 vs. 16/17 | 1.37 | 1.20 | 1.55 |
| 2017 vs. 2016 | 1.02 | 0.93 | 1.11 |
| 2018 vs. 2017 | 1.16 | 1.06 | 1.27 |
| 2019 vs. 2018 | 1.00 | 0.91 | 1.09 |
| **Level 3** |  |  |  |
| 18/19 vs. 16/17 | 0.84 | 0.68 | 1.03 |
| 2017 vs. 2016 | 0.94 | 0.81 | 1.08 |
| 2018 vs. 2017 | 0.92 | 0.80 | 1.07 |
| 2019 vs. 2018 | 1.05 | 0.90 | 1.22 |
| **Level 2** |  |  |  |
| 18/19 vs. 16/17 | 1.01 | 0.88 | 1.15 |
| 2017 vs. 2016 | 0.94 | 0.85 | 1.03 |
| 2018 vs. 2017 | 1.04 | 0.94 | 1.14 |
| 2019 vs. 2018 | 1.00 | 0.91 | 1.10 |
| **Level 1** |  |  |  |
| 18/19 vs. 16/17 | 0.74 | 0.65 | 0.84 |
| 2017 vs. 2016 | 1.01 | 0.92 | 1.10 |
| 2018 vs. 2017 | 0.86 | 0.78 | 0.94 |
| 2019 vs. 2018 | 1.00 | 0.91 | 1.10 |
| **Level 0** |  |  |  |
| 18/19 vs. 16/17 | 1.04 | 0.88 | 1.23 |
| 2017 vs. 2016 | 1.09 | 0.97 | 1.22 |
| 2018 vs. 2017 | 1.00 | 0.89 | 1.12 |
| 2019 vs. 2018 | 0.96 | 0.85 | 1.07 |
| Abbreviation: OR, odds ratio; CI, confidence interval; Level 0: <2 HbA1c tests within one year; Level1: ≥2 HbA1c tests within one year; Level 2: Level 1 and annual lipid profile; Level 3: Level 2 and annual nephropathy status; Level 4: Level 3 and visit to an ophthalmologist within one year | | | |
