# Supplementary material for: Does the implementation of an incentive scheme increase adherence to diabetes guidelines? A retrospective cohort study of managed care enrollees
Source: BMC Health Serv Res. 2023 Jun 29;23:707. doi: 10.1186/s12913-023-09694-z (PMC10308744; doi:10.1186/s12913-023-09694-z)
Supplement: Supplementary file 2 — Additional file 2. Predicted probability of receiving diabetesperformance measure after implementation of incentive scheme. N=6273. [file 12913_2023_9694_MOESM2_ESM.docx]

**Additional File 2**

Additional file 2: Predicted probability of receiving diabetes performance measure after implementation of incentive scheme

| **Performance measure** |  |  |  |
| --- | --- | --- | --- |
| **HbA1c test** | **OR** | **95% CI** | |
| 18/19 vs. 16/17 | 0.96 | 0.81 | 1.13 |
| 2017 vs. 2016 | 0.92 | 0.82 | 1.03 |
| 2018 vs. 2017 | 1.00 | 0.89 | 1.12 |
| 2019 vs. 2018 | 1.04 | 0.93 | 1.17 |
| **Total cholesterol test** |  |  |  |
| 18/19 vs. 16/17 | 1.19 | 1.06 | 1.35 |
| 2017 vs. 2016 | 0.95 | 0.87 | 1.03 |
| 2018 vs. 2017 | 1.15 | 1.05 | 1.25 |
| 2019 vs. 2018 | 0.96 | 0.88 | 1.04 |
| **HDL cholesterol test** |  |  |  |
| 18/19 vs. 16/17 | 1.58 | 1.40 | 1.78 |
| 2017 vs. 2016 | 1.01 | 0.93 | 1.10 |
| 2018 vs. 2017 | 1.24 | 1.14 | 1.35 |
| 2019 vs. 2018 | 1.01 | 0.92 | 1.10 |
| **LDL cholesterol test** |  |  |  |
| 18/19 vs. 16/17 | 1.14 | 0.97 | 1.34 |
| 2017 vs. 2016 | 0.98 | 0.87 | 1.09 |
| 2018 vs. 2017 | 1.11 | 0.99 | 1.24 |
| 2019 vs. 2018 | 0.95 | 0.85 | 1.07 |
| **Triglyceride test** |  |  |  |
| 18/19 vs. 16/17 | 1.27 | 1.13 | 1.43 |
| 2017 vs. 2016 | 0.98 | 0.90 | 1.07 |
| 2018 vs. 2017 | 1.15 | 1.06 | 1.25 |
| 2019 vs. 2018 | 0.98 | 0.90 | 1.07 |
| **Lipid profile test** |  |  |  |
| 18/19 vs. 16/17 | 1.27 | 1.12 | 1.43 |
| 2017 vs. 2016 | 0.98 | 0.90 | 1.06 |
| 2018 vs. 2017 | 1.15 | 1.05 | 1.25 |
| 2019 vs. 2018 | 0.98 | 0.90 | 1.07 |
| **Albuminuria test** |  |  |  |
| 18/19 vs. 16/17 | 1.18 | 1.05 | 1.33 |
| 2017 vs. 2016 | 1.09 | 1.00 | 1.18 |
| 2018 vs. 2017 | 1.00 | 0.92 | 1.09 |
| 2019 vs. 2018 | 1.08 | 1.00 | 1.18 |
| **Serum creatinine test** | |  |  |
| 18/19 vs. 16/17 | 1.35 | 1.16 | 1.57 |
| 2017 vs. 2016 | 1.00 | 0.90 | 1.11 |
| 2018 vs. 2017 | 1.20 | 1.08 | 1.33 |
| 2019 vs. 2018 | 0.94 | 0.84 | 1.05 |
| **Nephropathy status test** | |  |  |
| 18/19 vs. 16/17 | 1.20 | 1.07 | 1.35 |
| 2017 vs. 2016 | 1.08 | 1.00 | 1.18 |
| 2018 vs. 2017 | 1.03 | 0.95 | 1.12 |
| 2019 vs. 2018 | 1.05 | 0.97 | 1.14 |
| **Ophthalmologist visit** | |  |  |
| 18/19 vs. 16/17 | 1.34 | 1.13 | 1.59 |
| 2017 vs. 2016 | 1.08 | 0.96 | 1.21 |
| 2018 vs. 2017 | 1.16 | 1.03 | 1.30 |
| 2019 vs. 2018 | 0.93 | 0.82 | 1.04 |
| Abbreviation: OR, odds ratio; CI, confidence interval; HbA1c hemoglobin A1c; HDL, high-density lipoprotein; LDL, low-density lipoprotein | | | |
